# An ace up their sleeve: a transcriptomic approach exposes the AceI eﬄux protein of *Acinetobacter baumannii* and reveals the drug eﬄux potential hidden in many microbial pathogens

**DOI:** 10.3389/fmicb.2015.00333

**Published:** 2015-04-22

**Authors:** Karl A. Hassan, Liam D. H. Elbourne, Liping Li, Hasinika K. A. H. Gamage, Qi Liu, Scott M. Jackson, David Sharples, Anne-Brit Kolstø, Peter J. F. Henderson, Ian T. Paulsen

**Affiliations:** ^1^Department of Chemistry and Biomolecular Sciences, Macquarie UniversitySydney, NSW, Australia; ^2^Astbury Centre for Structural Molecular Biology, School of Biomedical Sciences, University of LeedsLeeds, UK; ^3^Laboratory for Microbial Dynamics, Department of Pharmaceutical Biosciences, School of Pharmacy, University of OsloOslo, Norway

**Keywords:** multidrug eﬄux systems, bacterial transmembrane pair, adaptive resistance, bacterial drug resistance transcriptomics

## Abstract

The era of antibiotics as a cure-all for bacterial infections appears to be coming to an end. The emergence of multidrug resistance in many hospital-associated pathogens has resulted in “superbugs” that are effectively untreatable. Multidrug eﬄux pumps are well known mediators of bacterial drug resistance. Genome sequencing efforts have highlighted an abundance of putative eﬄux pump genes in bacteria. However, it is not clear how many of these pumps play a role in antimicrobial resistance. Eﬄux pump genes that participate in drug resistance can be under tight regulatory control and expressed only in response to substrates. Consequently, changes in gene expression following antimicrobial shock may be used to identify eﬄux pumps that mediate antimicrobial resistance. Using this approach we have characterized several novel eﬄux pumps in bacteria. In one example we recently identified the *Acinetobacter* chlorhexidine eﬄux protein (AceI) eﬄux pump in *Acinetobacter*. AceI is a prototype for a novel family of multidrug eﬄux pumps conserved in many proteobacterial lineages. The discovery of this family raises the possibility that additional undiscovered intrinsic resistance proteins may be encoded in the core genomes of pathogenic bacteria.

## Introduction

**FIGURE 1:**
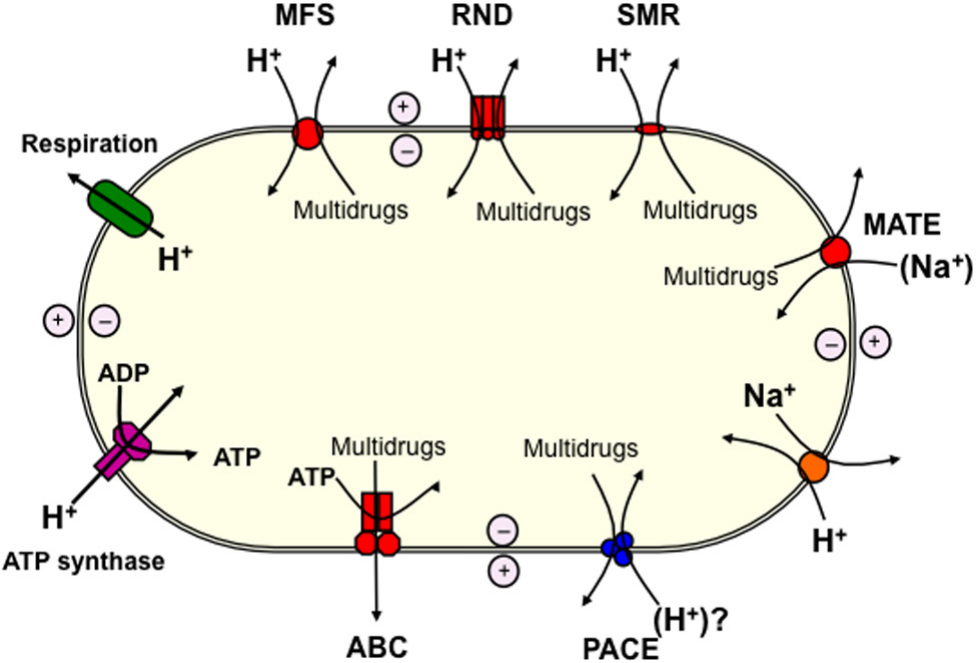
**Schematic diagram showing the basis for the energisation of multidrug eﬄux pumps operating in bacteria.** The large oval represents the bacterial cell. An electrochemical H^+^ gradient (proton-motive-force) across the cytoplasmic membrane is generated as a result of respiration (green). Energy from the proton-motive-force is used to power transport in secondary active transport systems, such as those within the major facilitator superfamily (MFS), resistance/nodulation/division (RND), and small multidrug resistance (SMR) (super) families. Sodium/proton antiporters (orange) harness the proton-motive-force to generate a Na^+^ gradient that powers transport by other multidrug eﬄux pumps, including those in the multidrug and toxic compound extrusion (MATE) family. ATP production by ATP-synthase (pink) is also powered by the proton-motive-force, and ATP is used to power transport by the primary active transporters of the ATP-binding cassette (ABC) superfamily. Previously characterized eﬄux pump (super) families are shown in red, whereas the proteobacterial antimicrobial compound eﬄux (PACE) family is shown in blue. The PACE family transport proteins are likely to be powered by the H^+^ gradient.

Multidrug eﬄux pumps are a significant obstacle preventing the control of infections caused by pathogenic bacteria. Genes encoding these transporters have been found in all bacterial genomes sequenced, and the overexpression of just one can lead to the reduced efficacy of a range of structurally and mechanistically unrelated antimicrobials (Ren and Paulsen, 2007; Brzoska et al., 2013). Five families of transporters that include multidrug eﬄux systems have been studied extensively, and include representative proteins that have been characterized biochemically and by tertiary structural analyses. These include the ATP-binding cassette (ABC) superfamily, the major facilitator superfamily (MFS), the resistance/nodulation/division (RND) superfamily, the small multidrug resistance (SMR) family, and the multidrug and toxic compound extrusion (MATE) family (**Figure 1**).

Significant longstanding difficulties surround identifying the physiological functions of these multidrug eﬄux transport proteins and determining which of the many pumps encoded by bacterial strains actually contribute to antimicrobial resistance (Piddock, 2006; Schindler et al., 2015). Studies have shown that these eﬄux pumps often have overlapping substrate recognition profiles (Tal and Schuldiner, 2009). Furthermore, it is not uncommon for a bacterial genome to encode a large number of eﬄux pumps that have predicted drug substrates, e.g., strains of *Bacillus cereus* encode more than 100 of these pumps accounting for more than 2% or their predicted protein coding potential (Ren and Paulsen, 2007; Simm et al., 2012). It is unlikely that all these pumps share the primary function of protection against toxic compounds, highlighting a need for higher throughput approaches to assess the physiological roles of individual proteins, be they in drug resistance, native housekeeping functions, or other cellular roles.

## Eﬄux Pumps Participate in Intrinsic, Adaptive, and Acquired Resistance

Bacterial drug resistance can be divided into three general categories, intrinsic, adaptive, and acquired (Fernandez and Hancock, 2012). Depending on their mode(s) of regulation and their local genetic context, bacterial multidrug eﬄux pumps can be geared to participate in any of these three resistance categories. Intrinsic resistance stems from inherent properties of a bacterial cell and can occur as a result of high constitutive expression and activity of some multidrug eﬄux pumps. Adaptive resistance is related to physiological alterations that are induced by environmental changes and can occur when multidrug eﬄux pumps are expressed in response to antimicrobial substrates. Finally, acquired resistance can result from mutations promoting constitutive expression of an ordinarily tightly controlled endogenous multidrug eﬄux system, or when eﬄux pump genes are acquired on a mobile genetic element, such as a plasmid or phage.

## Adaptive Resistance Responses Identify Eﬄux Pumps that Mediate Drug Resistance

High-level expression of eﬄux pumps can have a negative impact on cell growth (Brzoska et al., 2013), resulting in a need to control the timing of eﬄux system expression to coincide with specific physiological requirements. As such, eﬄux pumps with physiological resistance functions may be characteristically expressed in response to drug substrates. These pumps may be part of an adaptive drug resistance response or part of general stress response regulons. For example, expression of the *adeAB* and *adeIJK* eﬄux pump genes in *Acinetobacter baumannii* (Hassan et al., 2013; our unpublished data), the *acrAB* and *acrF* genes in *Escherichia coli* (Shaw et al., 2003; Bailey et al., 2009), the *mexXY* and *mexCD* genes in *Pseudomonas aeruginosa* (Morita et al., 2014), the *norA* gene in *Staphylococcus aureus* (Kaatz and Seo, 2004), and the *bmr* gene in *Bacillus subtilis* (Ahmed et al., 1994), is induced in response to antimicrobial shock treatments.

The mode of regulation and regulatory cues of most eﬄux pumps are typically only investigated after their functional characterisation. However, global gene expression profiles that show heightened expression of putative eﬄux pump genes following drug or toxin shocks have provided the impetus to assess the drug resistance functions of these pumps. For example, members of our team recognized that an uncharacterised MFS exporter, BC4707, was expressed in response to bile salt shock in the human food-poisoning associated pathogen *Bacillus cereus*, and went on to characterize its role in drug resistance (Kristoffersen et al., 2007). The gene encoding BC4707 is conserved in the core genome of *B. cereus* and its deletion from *B. cereus* ATCC 14579 resulted in increased susceptibility to norfloxacin (Simm et al., 2012). Overexpression of BC4707 in *E. coli* BL21 Δ*acrAB* resulted in increased resistance to norfloxacin, ciprofloxacin, and kanamyacin and fluorescence transport assays showed that accumulation of norfloxacin is reduced by BC4707 in an energy dependent manner (Simm et al., 2012).

## Adaptive Resistance Responses Identify a New Class of Drug Eﬄux Pump

Extending from this work, we have exploited adaptive resistance responses to identify eﬄux pumps that may mediate drug resistance in hospital-acquired bacterial pathogens, with a focus on biocide resistance. For example, in recent work we conducted a transcriptomic study to examine the regulatory response of *A. baumannii* to a shock treatment with the synthetic biocide chlorhexidine (Hassan et al., 2013). Chlorhexidine is commonly applied in antibacterial soaps, mouthwashes and antiseptics, and is listed as an “Essential Medicine” by the World Health Organization. Chlorhexidine is a membrane active biocide and as such, multidrug eﬄux pumps are commonly associated with reduced levels of susceptibility (Russell, 1986; McDonnell and Russell, 1999). In line with the discussion above, the most highly overexpressed genes in our chlorhexidine shock treatment encoded AdeAB, components of a major tripartite RND multidrug eﬄux system in *A. baumannii* (Hassan et al., 2013). This eﬄux system has previously been shown to mediate resistance to a very broad range of antimicrobials and biocides, including chlorhexidine (Rajamohan et al., 2009). The overexpression of genes encoding AdeAB in response to chlorhexidine confirmed the role of this eﬄux system in adaptive resistance to chlorhexidine in *A. baumannii*. Apart from the genes encoding AdeAB, only one gene was highly (>10-fold) overexpressed in response to chlorhexidine. This gene was originally annotated as encoding a hypothetical membrane protein. Using biochemical approaches we showed that this protein is in fact a chlorhexidine resistance protein that functions via an active eﬄux mechanism (Hassan et al., 2013). We named this protein the *Acinetobacter* chlorhexidine eﬄux protein I (AceI).

## The AceI Transporter is a Prototype for a New Family of Bacterial Multidrug Eﬄux Systems

The AceI transport protein contains two tandem “Bacterial Transmembrane Pair” (BTP) protein domains defined within the Pfam database (Finn et al., 2014). There are more than 750 protein sequences containing this domain architecture listed in the Pfam database (version 27.0). Genes encoding these proteins are particularly common among proteobacterial lineages, but can also be found in the genomes of unrelated bacterial genera, including the Firmicutes and Actinobacteria. We have not yet identified these genes in the genomes of any archaeal or eukaryotic organisms.

We have recently characterized more than 20 homologs of the AceI transporter by heterologous expression in *E. coli* (Hassan et al., 2015). These studies have demonstrated that many AceI homologs are able to provide resistance to an array of biocides in addition to chlorhexidine. For example, the VP1155 protein from *Vibrio parahaemolyticus* and Bcen2424_2356 protein from *Burkholderia cenocepacia* each conferred increased resistance to chlorhexidine, benzalkonium, acriflavine, and proflavine, when expressed in *E. coli* (Hassan et al., 2015). Fluorescence transport assays conducted on cells expressing these and other AceI homologs that conferred resistance to acriflavine and proflavine, demonstrated that these compounds are actively exported from the cell by these transporters (Hassan et al., 2015). These results corroborate our earlier findings that chlorhexidine is actively transported by AceI (Hassan et al., 2013), and indicate that eﬄux is the mechanism of resistance operating in this group of proteins. Taken together all the observations suggest that these proteins comprise a new family of multidrug eﬄux pumps common amongst Proteobacterial lineages. We have named this family the Proteobacterial Antimicrobial Compound Eﬄux (PACE) family (**Figure 1**; Hassan et al., 2015).

## PACE Family Proteins are Encoded Within the Core Genome

Given that the PACE family represents a new class of resistance determinants, we were interested in gathering basic information regarding the mode of inheritance of these genes in bacteria. To this end, we examined their level of conservation within representative bacterial lineages following the basic premise that highly conserved genes within core bacterial genomes are expected to have been inherited vertically, whereas those in the accessory genome are likely to have been horizontally acquired.

We examined PACE family protein conservation in four γ-Proteobacterial species (*A. baumannii*, *P. aeruginosa*, *V. parahaemolyticus,* and *E. coli*) a β-Proteobacterial species (*B. cenocepacia*) and a member of the Firmicutes (*Veillonella parvula*). Annotated protein sequences from all complete and draft genomes of these species were downloaded from the NCBI Genbank database (October, 2014) and were queried using the BTP PfamHMM (Finn et al., 2014) in HMMER3 searches (Eddy, 2011). These searches determined that PACE family proteins are encoded in the pan-genomes of all six species examined. To determine the number of distinct orthologous groups of PACE family proteins in each species we performed a clustering analysis based on sequence identity (cluster stringency >90%) using cd-hit v4.6.1 (Fu et al., 2012). This analysis demonstrated that *A. baumannii* had three clusters (100, 96.7, and 0.3% conservation in 623 strains); *P. aeruginosa* had three clusters (99.5, 99.5, and 0.5% conservation in 197 strains); *V. parahaemolyticus* had one cluster (90.1% conservation in 101 strains); *E. coli* had 4 clusters (0.2, 0.1, 0.1, and 0.1% conservation in 1986 strains); *B. cenocepacia* had three clusters (100, 88.9, and 88.9% conservation in nine strains); and *V. parvula* had one cluster (100% conservation in four strains).

These data demonstrate that the pattern of PACE family protein conservation is variable between the species. For example, both *A. baumannii* and *P. aeruginosa* each encode two highly conserved PACE family proteins present in virtually all sequenced strains, and one additional PACE protein encoded in one or two specific strains. Whereas, *V. parahaemolyticus* and *V. parvula* each encode only one highly conserved PACE protein, and *B. cenocepacia* encodes three highly conserved PACE proteins. Most *E. coli* strains do not encode a PACE family protein, although a small handful of strains encode one of four PACE protein variants. The highly conserved PACE family proteins encoded by *A. baumannii*, *P. aeruginosa*, *V. parahaemolyticus*, *B. cenocepacia,* and *V. parvula* are likely to constitute part of the core genome in these species and to have been inherited vertically rather than on mobile genetic elements. The almost complete lack of genes encoding PACE family proteins in *E. coli* strains suggests that these genes were lost early in the development of the *E. coli* lineage, but after its divergence from other γ-proteobacteria. In the few cases where *E. coli* strains were found to encode a PACE family protein, it was sometimes associated with mobile genetic elements suggesting that it had been acquired by horizontal gene transfer. The paucity of PACE genes in *E. coli* strains confirms our previous conclusion that *E. coli* is an excellent host to study the function of these proteins (Hassan et al., 2013).

## Physiological Substrates for PACE Family Transporters

To date, the substrates identified for PACE family transport proteins include synthetic biocides only, such as chlorhexidine, dequalinium, benzalkonium, proflavine, and acriflavine. The presence of these toxic biocides in the environments occupied by Proteobacteria is likely to have been negligible across evolutionary time, until perhaps the last 50–100 years when these compounds were applied in various industries. Given that the organisms encoding PACE family genes are likely to have diverged long before the development of this potential selective pressure, it is seems unlikely that biocides are the native physiological substrates of PACE eﬄux pumps. Nonetheless, these genes are transcriptionally responsive to at least one biocide, chlorhexidine in four species, *A. baumannii*, *A. baylyi*, *P. aeruginosa,* and *B. cenocepacia* (Nde et al., 2009; Coenye et al., 2011; Hassan et al., 2013), suggesting that chlorhexidine can serve as a mimic of their natural physiological substrate for inducing eﬄux pump expression.

## Regulatory Proteins Acting on PACE Eﬄux Pumps

In addition to antimicrobial resistance, the promiscuous substrate recognition profiles of multidrug eﬄux pumps allow them to participate in diverse physiological processes. For example, eﬄux systems in Gram-negative bacteria function in cell adherence, invasion, biofilm formation, virulence in animals and plants, and resistance to host encoded factors (Piddock, 2006). Consequently, the regulation of bacterial drug eﬄux systems can be highly complex and responsive to a range of cellular and extracellular conditions. Complex regulation may be particularly apparent in eﬄux pumps, such as AceI and its homologs, which are encoded within core bacterial genomes. These genes are likely to have been present in these species for significant periods of evolutionary time, allowing fine-tuning of their expression in response to a range of environmental cues. A case in point, as summarized within the EcoCyc database (Keseler et al., 2013), transcription of the *acrAB* eﬄux system genes, within the core genome of *E. coli*, is controlled by at least seven distinct regulatory proteins, which are themselves subject to a range of regulatory pressures. These regulatory proteins are likely to integrate eﬄux pumps into the adaptive resistance responses observed in bacteria, as well as other pathways controlling their alternative physiological functions.

Regulators mediating the most direct control of genes encoding eﬄux pumps are often encoded locally – adjacent to and divergently transcribed from the gene(s) encoding the eﬄux system. These regulators, either activators or repressors, typically bind a similar spectrum of compounds to their cognate eﬄux pump with high affinity as a signal for transcriptional activation or relief of transcriptional repression. Some well characterized examples include AcrR, which controls transcription of the *E. coli acrAB* eﬄux pump genes (Li et al., 2007), and QacR, which controls *qacA*/*qacB* expression in *S. aureus* (Grkovic et al., 1998; Schumacher et al., 2001).

**FIGURE 2:**
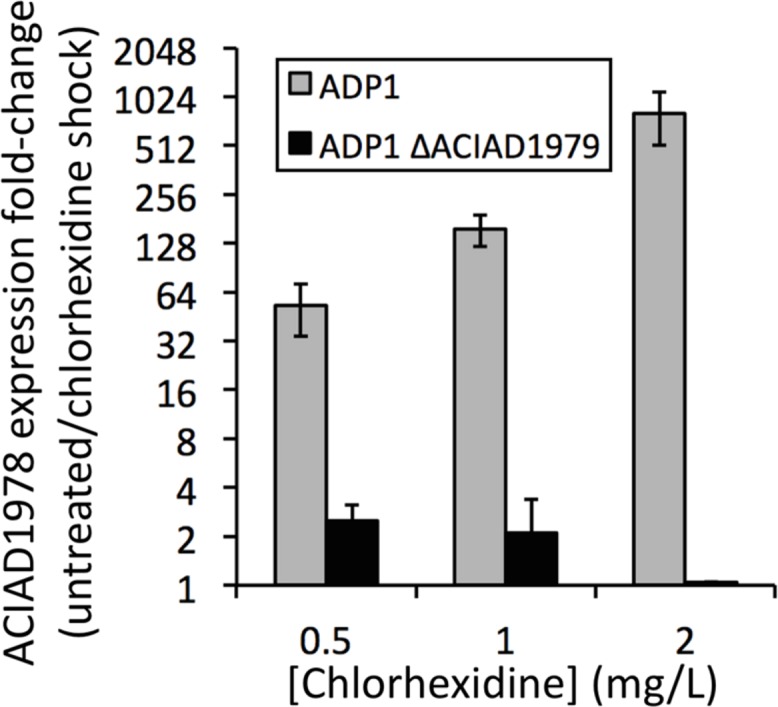
**Expression of the PACE family gene, ACIAD1978, in wild-type and mutant (ΔACIAD1979) *A. baylyi* ADP1 in response to chlorhexidine shock treatments.** Cells were grown in LB broth to OD_600_ = 0.6, then split, with one half of each culture treated with chlorhexidine and the other half receiving no treatment. RNA was isolated and assessed by qRT-PCR following our established protocols (Brzoska and Hassan, 2014) to determine relative expression levels of ACIAD17978 in chlorhexidine treated and untreated samples. Error bars show the SEM. Changes in expression of ACIAD1978 were negligible in the ACIAD1979 mutant treated with 2 mg/L chlorhexidine, and thus the bar is difficult to see.

The PACE family transporters that we have studied to date are each encoded adjacent to a divergently transcribed LysR family regulator. To determine whether these regulators control the expression of their cognate PACE family gene, we used our established methods (Brzoska et al., 2013) to construct a deletion mutant of the regulator gene ACIAD1979 in *A. baylyi* ADP1, which is encoded adjacent to the PACE family chlorhexidine resistance gene ACIAD1978. We examined the expression of ACIAD1978 in both the wild-type and the ΔACIAD1979 regulatory mutant in response to chlorhexidine shock treatments using quantitative real-time PCR analysis (Brzoska and Hassan, 2014). In the absence of chlorhexidine the expression of ACIAD1978 was similar in both strains. However, whereas increasing concentrations of chlorhexidine induced ACIAD1978 gene expression in the wild-type strain, chlorhexidine addition failed to induce ACIAD1978 expression in the ΔACIAD1979 mutant (**Figure 2**). These results suggest that the ACIAD1979 LysR family regulator functions as an activator of the PACE family gene ACIAD1978. We are currently investigating the role of LysR family proteins in controlling expression of PACE family pumps in other species and are determining whether the spectrum of ligands recognized by these regulators is closely linked to the substrate recognition profile of their cognate PACE family pump. It also remains to be determined whether the PACE-associated regulators control expression of other genes, or if there are distally encoded regulators that also modulate expression of PACE transporter genes.

## Conclusion and Future Directions

Transcriptomic analyses of antimicrobial shock treatments are valuable in identifying the potential resistance mechanisms operating in bacteria, including multidrug eﬄux pumps participating in the adaptive resistance response. Using transcriptomic analyses, we have defined roles for new eﬄux pumps and identified the PACE family of multidrug transport proteins, the first new family of drug eﬄux proteins discovered in over a decade.

Transporters within the PACE family are currently enigmas. We have identified drug substrates, such as chlorhexidine that are common to many of these pumps. Furthermore, PACE family gene expression is induced by chlorhexidine, a response that is mediated via locally encoded regulators. This highlights a close relationship between the function of these pumps and their regulatory control. Since PACE family genes are encoded in the core genomes of bacterial lineages that diverged long ago, this functional-regulatory relationship is likely to have arisen early in the evolution of these proteins. However, the substrates/inducers that have been identified for PACE proteins are synthetic biocides that are likely to have been absent from the environment until the last 50–100 years. Therefore, it is unlikely that these biocides would have provided the selective pressure required to drive the functional or regulatory evolution of PACE family pumps. Consequently, a deeper understanding of these novel resistance proteins requires future investigations aimed at identifying their physiological substrate(s) and primary functional roles in bacteria.

The discovery of the PACE family opens up the possibility that there may be more novel eﬄux proteins waiting to be discovered. There are many hypothetical membrane proteins of unknown function encoded in all bacterial genomes. For example, even in the best-studied bacterial genome, *E. coli* K12, there are 409 membrane proteins of unknown function. At least some of these may represent entirely novel types of eﬄux pumps.

## Conflict of Interest Statement

The authors declare that the research was conducted in the absence of any commercial or financial relationships that could be construed as a potential conflict of interest.

## References

[B1] Ahmed M. Borsch C. M. Taylor S. S. Vázquez-Laslop N. Neyfakh A. A. (1994). A protein that activates expression of a multidrug eﬄux transporter upon binding the transporter substrates. *J. Biol. Chem.* 269 28506–28513. 7961792

[B2] Bailey A. M. Constantinidou C. Ivens A. Garvey M. I. Webber M. A. Coldham N. (2009). Exposure of *Escherichia coli* and *Salmonella enterica* serovar typhimurium to triclosan induces a species-specific response, including drug detoxification. *J. Antimicrob. Chemother.* 64 973–985 10.1093/jac/dkp320 19759044

[B3] Brzoska A. J. Hassan K. A. (2014). Quantitative PCR for detection of mRNA and gDNA in environmental isolates. *Methods Mol. Biol.* 1096 25–42 10.1007/978-1-62703-712-9_3 24515358

[B4] Brzoska A. J. Hassan K. A. De Leon E. J. Paulsen I. T. Lewis P. J. (2013). Single-step selection of drug resistant *Acinetobacter baylyi* ADP1 mutants reveals a functional redundancy in the recruitment of multidrug eﬄux systems. *PLoS ONE* 8:e56090 10.1371/journal.pone.0056090 PMC3567077 23409126

[B5] Coenye T. Van Acker H. Peeters E. Sass A. Buroni S. Riccardi G. (2011). Molecular mechanisms of chlorhexidine tolerance in *Burkholderia cenocepacia* biofilms. *Antimicrob. Agents Chemother.* 55 1912–1919 10.1128/AAC.01571-10 21357299 PMC3088199

[B6] Eddy S. R. (2011). Accelerated profile HMM searches. *PLoS Comput. Biol.* 7:e1002195 10.1371/journal.pcbi.1002195 PMC3197634 22039361

[B7] Fernandez L. Hancock R. E. (2012). Adaptive and mutational resistance: role of porins and eﬄux pumps in drug resistance. *Clin. Microbiol. Rev.* 25 661–681 10.1128/CMR.00043-12 23034325 PMC3485749

[B8] Finn R. D. Bateman A. Clements J. Coggill P. Eberhardt R. Y. Eddy S. R. (2014). Pfam: the protein families database. *Nucleic. Acids Res.* 42 D222–D230 10.1093/nar/gkt1223 24288371 PMC3965110

[B9] Fu L. Niu B. Zhu Z. Wu S. Li W. (2012). CD-HIT: accelerated for clustering the next-generation sequencing data. *Bioinformatics* 28 3150–3152 10.1093/bioinformatics/bts565 23060610 PMC3516142

[B10] Grkovic S. Brown M. H. Roberts N. J. Paulsen I. T. Skurray R. A. (1998). QacR is a repressor protein that regulates expression of the *Staphylococcus aureus* multidrug eﬄux pump QacA. *J. Biol. Chem.* 273 18665–18673 10.1074/jbc.273.29.18665 9660841

[B11] Hassan K. A. Jackson S. M. Penesyan A. Patching S. G. Tetu S. G. Eijkelkamp B. A. (2013). Transcriptomic and biochemical analyses identify a family of chlorhexidine eﬄux proteins. *Proc. Natl. Acad. Sci. U.S.A*. 110 20254–20259 10.1073/pnas.1317052110 24277845 PMC3864336

[B12] Hassan K. A. Li Q. Henderson P. J. F. Paulsen I. T. (2015). Homologs of the *Acinetobacter baumannii* aceI transporter represent a new family of bacterial multidrug eﬄux systems. *mBio* 6 e01982–e01914 10.1128/mBio.01982-14 25670776 PMC4337561

[B13] Kaatz G. W. Seo S. M. (2004). Effect of substrate exposure and other growth condition manipulations on *norA* expression. *J. Antimicrob. Chemother.* 54 364–369 10.1093/jac/dkh341 15231765

[B14] Keseler I. M. Mackie A. Peralta-Gil M. Santos-Zavaleta A. Gama-Castro S. Bonavides-Martinez C. (2013). EcoCyc: fusing model organism databases with systems biology. *Nucleic. Acids Res.* 41 D605–D612 10.1093/nar/gks1027 23143106 PMC3531154

[B15] Kristoffersen S. M. Ravnum S. Tourasse N. J. Okstad O. A. Kolsto A. B. Davies W. (2007). Low concentrations of bile salts induce stress responses and reduce motility in *Bacillus cereus* ATCC 14579. *J. Bacteriol.* 189 5302–5313 10.1128/JB.00239-07 17496091 PMC1951874

[B16] Li M. Gu R. Su C. C. Routh M. D. Harris K. C. Jewell E. S. (2007). Crystal structure of the transcriptional regulator AcrR from *Escherichia coli*. *J. Mol. Biol.* 374 591–603 10.1016/j.jmb.2007.09.064 17950313 PMC2254304

[B17] McDonnell G. Russell A. D. (1999). Anitseptics and disinfectants: activity, action and resistance. *Clin. Microbiol. Rev.* 12 147–179. 9880479 10.1128/cmr.12.1.147 PMC88911

[B18] Morita Y. Tomida J. Kawamura Y. (2014). Responses of *Pseudomonas aeruginosa* to antimicrobials. *Front. Microbiol.* 4:422 10.3389/fmicb.2013.00422 PMC3884212 24409175

[B19] Nde C. W. Jang H. J. Toghrol F. Bentley W. E. (2009). Global transcriptomic response of *Pseudomonas aeruginosa* to chlorhexidine diacetate. *Environ. Sci. Technol.* 43 8406–8415 10.1021/es9015475 19924977

[B20] Piddock L. J. (2006). Multidrug-resistance eﬄux pumps - not just for resistance. *Nat. Rev. Microbiol.* 4 629–636 10.1038/nrmicro1464 16845433

[B21] Rajamohan G. Srinivasan V. B. Gebreyes W. A. (2009). Novel role of *Acinetobacter baumannii* RND eﬄux transporters in mediating decreased susceptibility to biocides. *J. Antimicrob. Chemother.* 65 228–232 10.1093/jac/dkp427 20008046

[B22] Ren Q. Paulsen I. T. (2007). Large-scale comparative genomic analyses of cytoplasmic membrane transport systems in prokaryotes. *J. Mol. Microbiol. Biotechnol.* 12 165–179 10.1159/000099639 17587866

[B23] Russell A. D. (1986). Chlorhexidine: antibacterial action and bacterial resistance. *Infection* 14 212–215 10.1007/BF01644264 3539812

[B24] Schindler B. D. Frempong-Manso E. Demarco C. E. Kosmidis C. Matta V. Seo S. M. (2015). Analyses of multidrug eﬄux pump-like proteins encoded on the *Staphylococcus aureus* chromosome. *Antimicrob. Agents Chemother.* 59 747–748 10.1128/AAC.04678-14 25403665 PMC4291402

[B25] Schumacher M. A. Miller M. C. Grkovic S. Brown M. H. Skurray R. A. Brennan R. G. (2001). Structural mechanisms of QacR induction and multidrug recognition. *Science* 294 2158–2163 10.1126/science.1066020 11739955

[B26] Shaw K. J. Miller N. Liu X. Lerner D. Wan J. Bittner A. (2003). Comparison of the changes in global gene expression of *Escherichia coli* induced by four bactericidal agents. *J. Mol. Microbiol. Biotechnol.* 5 105–122 10.1159/000069981 12736533

[B27] Simm R. Voros A. Ekman J. V. Sodring M. Nes I. Kroeger J. K. (2012). BC4707 is a major facilitator superfamily multidrug resistance transport protein from *Bacillus cereus* implicated in fluoroquinolone tolerance. *PLoS ONE* 7:e36720 10.1371/journal.pone.0036720 PMC3353944 22615800

[B28] Tal N. Schuldiner S. (2009). A coordinated network of transporters with overlapping specificities provides a robust survival strategy. *Proc. Natl. Acad. Sci. U.S.A.* 106 9051–9056 10.1073/pnas.0902400106 19451626 PMC2690002

